# Artificial intelligence for localization of the acute ischemic stroke by non-contrast computed tomography

**DOI:** 10.1371/journal.pone.0277573

**Published:** 2022-12-01

**Authors:** Natsuda Kaothanthong, Kamin Atsavasirilert, Soawapot Sarampakhul, Pantid Chantangphol, Dittapong Songsaeng, Stanislav Makhanov

**Affiliations:** 1 Sirindhorn International Institute of Technology, Thammasat University, Pathum Thani, Thailand; 2 Division of Diagnostic Radiology, Department of Radiology, Faculty of Medicine, Siriraj Hospital, Mahidol University, Bangkok, Thailand; Vellore Institute of Technology: VIT University, INDIA

## Abstract

A non-contrast cranial computer tomography (ncCT) is often employed for the diagnosis of the early stage of the ischemic stroke. However, the number of false negatives is high. More accurate results are obtained by an MRI. However, the MRI is not available in every hospital. Moreover, even if it is available in the clinic for the routine tests, emergency often does not have it. Therefore, this paper proposes an end-to-end framework for detection and segmentation of the brain infarct on the ncCT. The computer tomography perfusion (CTp) is used as the ground truth. The proposed ensemble model employs three deep convolution neural networks (CNNs) to process three end-to-end feature maps and a hand-craft features characterized by specific contra-lateral features. To improve the accuracy of the detected infarct area, the spatial dependencies between neighboring slices are employed at the postprocessing step. The numerical experiments have been performed on 18 ncCT-CTp paired stroke cases (804 image-pairs). The leave-one-out approach is applied for evaluating the proposed method. The model achieves 91.16% accuracy, 65.15% precision, 77.44% recall, 69.97% F1 score, and 0.4536 IoU.

## Introduction

The ischemic brain stroke occurs when the blood supply to a part of the brain is reduced by the blood clots. The accurate detection of the stroke is usually established by an MRI [1–7]. However, the MRI is not available in every hospital. Moreover, even when it is available in the clinic for the routine tests the Emergency often does not have it. The ncCT is used for an initial stroke diagnosis and for differentiating between the stroke subtypes such as the ischemic stroke, the haemorrhage, and the transient ischaemic attack. It should also be able to differentiate between the actual stroke and the stroke mimicking lesions [8, 9]. In order to improve the diagnosis, a contrast media is injected to obtain the Computer Tomography Angiography (CTA) or a Computer Tomography perfusion (CTp) [10].

Several automated imaging software have been developed to detect the acute ischemic stroke (AIS). They have been integrated into decision-making relevant to thrombectomy (the surgery to remove the clot in the blood vessel). An example is RAPID [11]. The software has been validated in several intervention trials. Over one thousand hospitals use the software in their clinical practice. The recent version includes the ASPECTS scoring module based on the machine learning. The Alberta Stroke Program Early Computed Tomography Score (ASPECTS) is a promising tool for the evaluation of stroke expansion to determine suitability of the available therapy. The ASPECTS is clinically used to subjectively assess extent of the early stroke using a 10 point segmental assessment of the middle cerebral artery [12]. As a semi-quantitative measure, ASPECTS, requires expertise and is observer dependent. However, recent research shows the evidence that ASPECTS can be assessed objectively with comparable accuracy to expert readings [13]. Olean [14] is another example of a commercially available software. Compared to RAPID, it has similar characteristics although verification on the MRI images shows a slight advantage of RAPID [11]. The final decision based on the contrast imagery still requires a radiologist experienced in processing these types of images. Besides, the visualization and classification software mentioned above is expensive. Therefore, reading of the ncCT by the radiologist remains the most common way of detection of the AIS [15]. The ncCT images have been used for the infarct segmentation by [16], while having the MRI as the ground truth. Fusion of the MRI images has been proposed to enhance the visibility of the infarct area on the ncCT images by [7]. Yahiaoui et al. [17] utilize a contrast enhancement method on ncCT images to segment the brain lesions of an ischemic stroke patients. Wu et al. propose a symmetry patch-based classification. They described an image patch in one brain hemisphere with its contralateral using a set of radiomic features [18]. The information from the neighboring patches is used to correct the final result. The segmentation of the ncCT images has been proposed for the poststroke to measure the brain damage by [19, 20]. The prediction of the ASPECTS, has also been performed on ncCT to evaluate the suitability of the thrombolysis (therapy of the AIS) [21]. The results have been validated by the MRI images.

We show that computerized image analysis developed using machine learning and artificial intelligence offers a fast, consistent and precise interpretation of ncCT for the assessment of the AIS [22]. The proposed end-to-end framework for detection and localization of the brain infarct area on the ncCT has been verified by the computer tomography perfusion (CTp) produced from RAPID software and used as the ground truth. The proposed ensemble model employs four features, where three are obtained from the different deep CNNs to process end-to-end feature maps and the last one is a contralateral hand-crafted feature. To improve the consistency of the detected infarct area, the spatial dependencies between neighboring slices are employed at the postprocessing step. Though many image processing algorithms deal with the infarct segmentation, as a rule they use the MRI or poststroke CT images as the ground truth. However, the poststroke MRI does not show the actual lesion since the brain damage is developing very fast (in a matter of minutes) once the stroke is onset. One of the promising research directions are the Convolution Neural Networks (CNNs) applied nCCT during the active phase of the AIS. If properly trained the CNNS be able to extract complex features which are impossible to hand-craft [23–26]. In the past the CNNs have been applied to diagnosing the brain stroke [3, 27–29], brain tumors [30], lung cancer [31], irregularities of the retina [32], and breast cancer [33]. CNNs have been also utilized for finding suitable perfusion parameter settings of CTp for segmenting the lesions [34].

Dilated convolutions is a module of the CNN that extracts features without losing the coverage of the receptive field [35]. This way, the features represent a wider view which improves the semantic segmentation. In addition, the end-to-end features are extracted by building a pixel-by-pixel mapping between the image’s content and the label [36]. The dilated convolution module is integrated in DRINet [37]. The result is compared to the manual labels obtained from experienced radiologists.

An ensemble model is a machine learning approach that combines multiple models in the prediction process to cope with a high variance of the input and bias of the features and overfitting. Our ensemble model is inspired by the automatic segmentation of the infarct in a follow-up ncCT [38]. However, as opposed to the above model which uses the poststroke imagery we use the images obtained when the diagnosis still has not been established. We solve the two basic problems of the ncCT based diagnosis i.e. defining the label of the CTp and dealing with the difference between the healthy and unhealthy tissues in the earl stage of the AIS. In order to differentiate between the healthy and infarct pixels the end-to-end features are extracted from each ncCT slice by a deep CNN and stored as the feature map. Following [38] a dilated CNN generates three different feature maps where each is extracted using different deep neural networks, namely, Mobile Net, ResNet50, and ResNet101. The three feature maps together with the pixel-wise features are fed to the proposed ensemble model in order to obtain a consistent infarct area between the consecutive slices.

## Previous work

### Brain infarct localization

**Fig 1:**
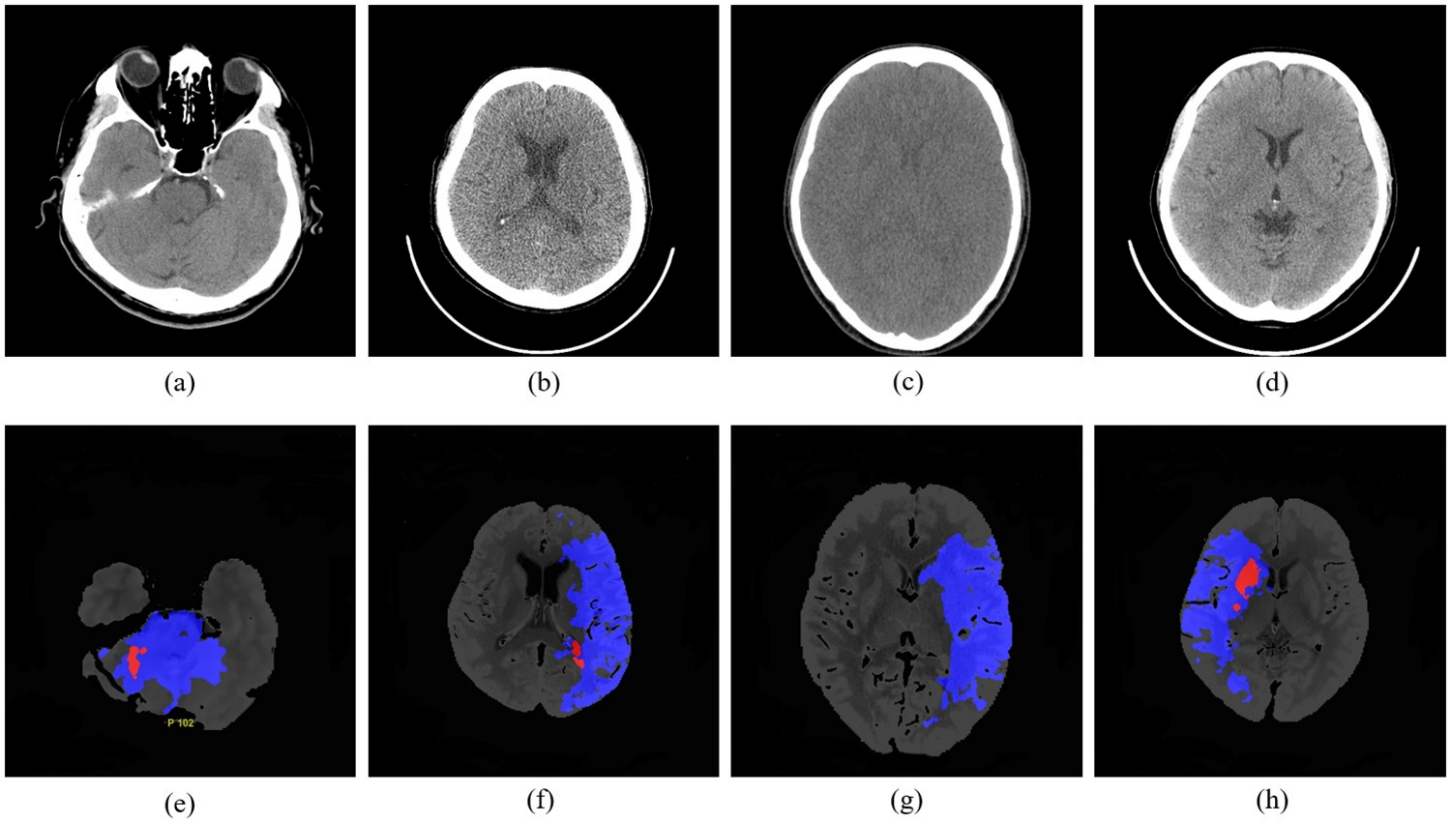
Examples of nCCT images, (a)-(d), and the corresponding CTp, (e)-(h). The color shows the cerebral blood volume (CBV), cerebral blood flow (CBF), the mean transit time (MTT) generated by CTp, the ischemic area- blue, the dead tissue-red.

Computed Tomography (CT) is a computerized X-ray imaging to produce cross-sectional images (slices). Each slice is a 2D image, where each pixel is represented by a Hounsfield Unit (HU) ranging from -1000 to 1000. [39] recommends the so-called stroke window 40-100 to map the HU onto the grey level. However, this procedure is not always applicable to the images of the early stage. Sim et al. [40] applies the central moment, mean, variance, kurtosis, and skewness to find a suitable setting. Lailatul Mugniroq [41] experiments with the different window size in the framework of detecting the subacute ischemic stroke. Przelaskowski et al. [42] introduces a perception method of the AIS based on the local contrast enhancement and a multi-scale approach to improve the visibility of the hypodense area. Hiroyuki Nagashima [43] uses the mA values to improve the quality of the image. CTp maps the volume of the blood flow in the brain to the color map [44]. Examples of ncCTs ((a)—(d)) and their corresponding CTps ((e)—(h)) are shown in Fig 1. The red color shows the dead tissue, whereas the blue color is the area with the low blood flow. The visualization of the CTp requires a pre-defined threshold to separate the brain damage levels. Flottmann et al. [45] presents a threshold-free method for the predicting brain infarct from CTp. The method solves the problem of mismatch between different patients caused by the thresholds.

Non-Contrast Computer Tomography (ncCT) and CT angiography (CTA) are extensively used for the diagnosis and treatment of AIS patients. To segment the brain area on ncCT and CTA, Najm et al. presents a method that approximates the contour of the segmented region using the segmented contour of the previous slice [46]. A number of approaches have been proposed for the AIS segmentation and prediction based on the MRI and CT [47]. However, the use of the ncCT is still limited. The deep neural network (DNN) architecture named U-net [48–50] applies CNN for feature extraction. It is extensively used for image segmentation in medical and biomedical image processing. In particular the recent papers on application of the U-net to CT-stroke segmentation are [51, 52].

### Semantic segmentation by DeepLabV3+

Chen et al. [53] demonstrates that the DNN DeepLabV3+ is able to capture the contextual information for a semantic segmentation using multiple scales of the extracted features. The DNN includes an encoder and a decoder combined with CNN Xception-65 [54] with the atrous convolution layers to get the coarse feature map. The encoder has three channels of the input for the image and its labels. The input size is gradually reduced to diversify extracted information. The decoder applies a conditional random field to produce a final output by recovering the spatial information of the encoded feature map.

The advantage of the DeepLabV3+ is a wider view of neighboring pixels when performing the feature extraction which is necessary for a semantic segmentation to understand the surrounding area in order to assign the same label to the same region. Besides, the CNNs architecture applied as a backbone can be changed. This way, a set of different features can be obtained.

## Methodology

**Fig 2:**
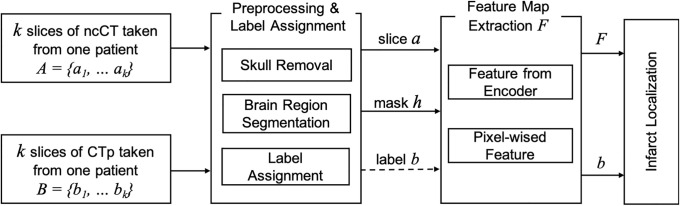
Three basic steps of the proposed model 1) label assignment, 2) feature map extraction, 3) infarct localization.

The proposed method has been illustrated in Fig 2. The preprocessing includes CTp-ncCT image registration and the pixel-wise labeling based on the CTp image. The next step produces features extracted by DeepLabv3+. This DNN uses selected backbone networks as well as hand-crafted pixel-wise features. The feature maps are then used by an ensemble model designed for localization and segmentation. The detected infarct area is evaluated using classification measures i.e. the accuracy, the precision and the recall (sensitivity). The precision shows the preciseness of the predicted infarct area as compared to the label on the CTp. The recall(sensitivity) measures how accurately the actual infarct area is detected. Besides, the intersection over union (IoU) is the ratio of the overlap of the detected area and the ground truth and the union of these two parameters. The classification measures are defined in section “Data set and performance evaluation”.

### Preprocessing and label assignment

**Fig 3:**
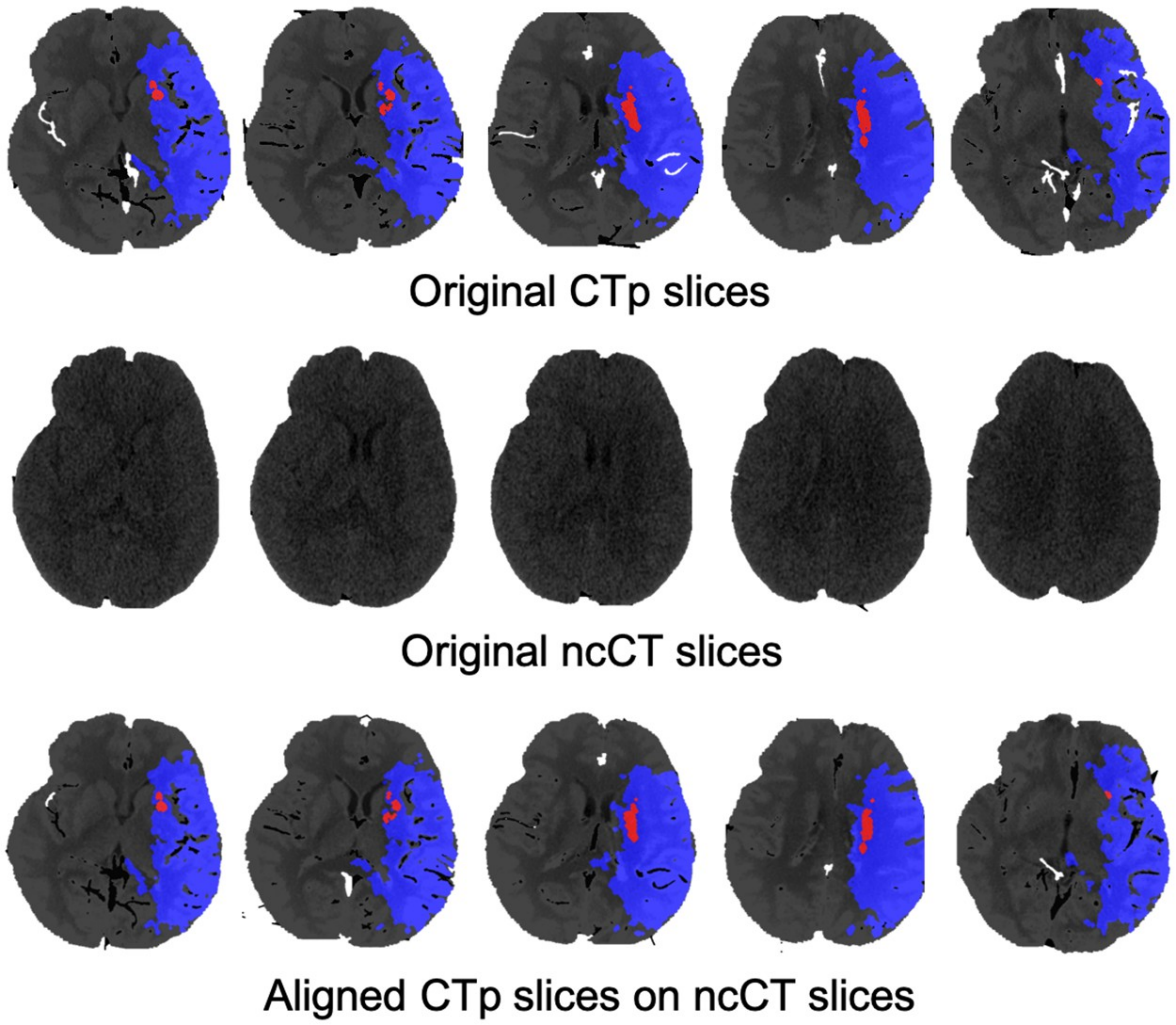
CTp with the ground truth, ncCT aligned with the CTp.

Note that the aspect ratio of CTp and ncCT is different. In order to align the images, translation, rotation, and scaling the CTp image is applied. Let *A* = {*a*_1_, …, *a*_*k*_} be a set of ncCT slices. Each slice is preprocessed by removing the skull and leaving the largest connected brain region. Further *H* = {*h*_1_, …, *h*_*k*_} denotes a set the images of the largest connected brain regions corresponding to *A*. Further, *B* = {*b*_1_, …, *b*_*k*_} is a set of the CTp slices. A pair *a*_*i*_, *b*_*i*_ is selected automatically or by a radiologist. Examples of such pairs are shown in Fig 3. The last row shows the CTp images with superimposed ncCT-ground truth. In order evaluate the transformation matrix to align the CTp- and ncCT, representative slices *a*_*i*_, *b*_*i*_ in the middle of the set are selected. Alternatively, such a pair is selected manually.

The alignment is performed by minimizing the difference between the corresponding convex hulls. Given the representative pair *a*_*i*_ and *b*_*i*_ consider a set of boundary points corresponding to *a*_*i*_ and *b*_*i*_. The corresponding convex hulls are denoted by *H*_*A*_ and *H*_*B*_. The alignment is performed by the translation *t*_*x*_, *t*_*y*_ and the rotation *θ* of *H*_*A*_ to minimize the difference between the area of *H*_*A*_ and *H*_*B*_. Examples of the alignment are shown in Fig 3. Each pixel in *b*_*i*_ is labeled the same way as the closest pixel in *a*_*i*_. The resulting transformation matrix is applied to every pair (*a*, *b*). Note that selecting the representative pair is used to reduce the computational time. The model is able to evaluate the corresponding transformation for each pair of the images individually.

### Feature map extraction

**Fig 4:**
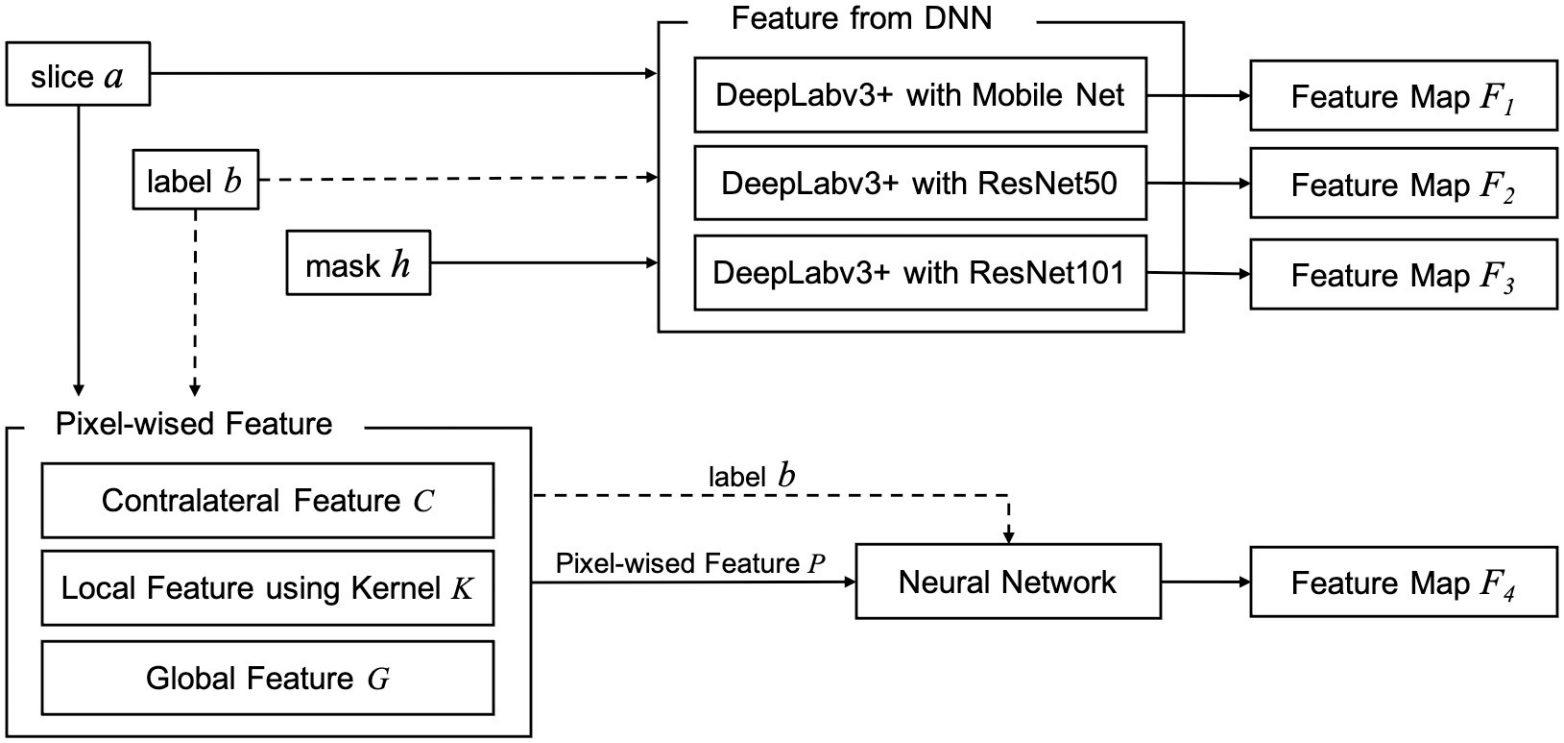
The architecture of the input for the proposed DNN.

The model generates the four feature maps {*F*_1_, *F*_2_, *F*_3_, *F*_4_}. The features {*F*_1_, *F*_2_, *F*_3_} are generated by deep neural network DeepLabv3+ [55]. *F*_4_ is a contralateral map based on the difference between the right and the left side of the image. The proposed procedure is depicted in Fig 4.

#### Feature maps by DNN

**Fig 5:**
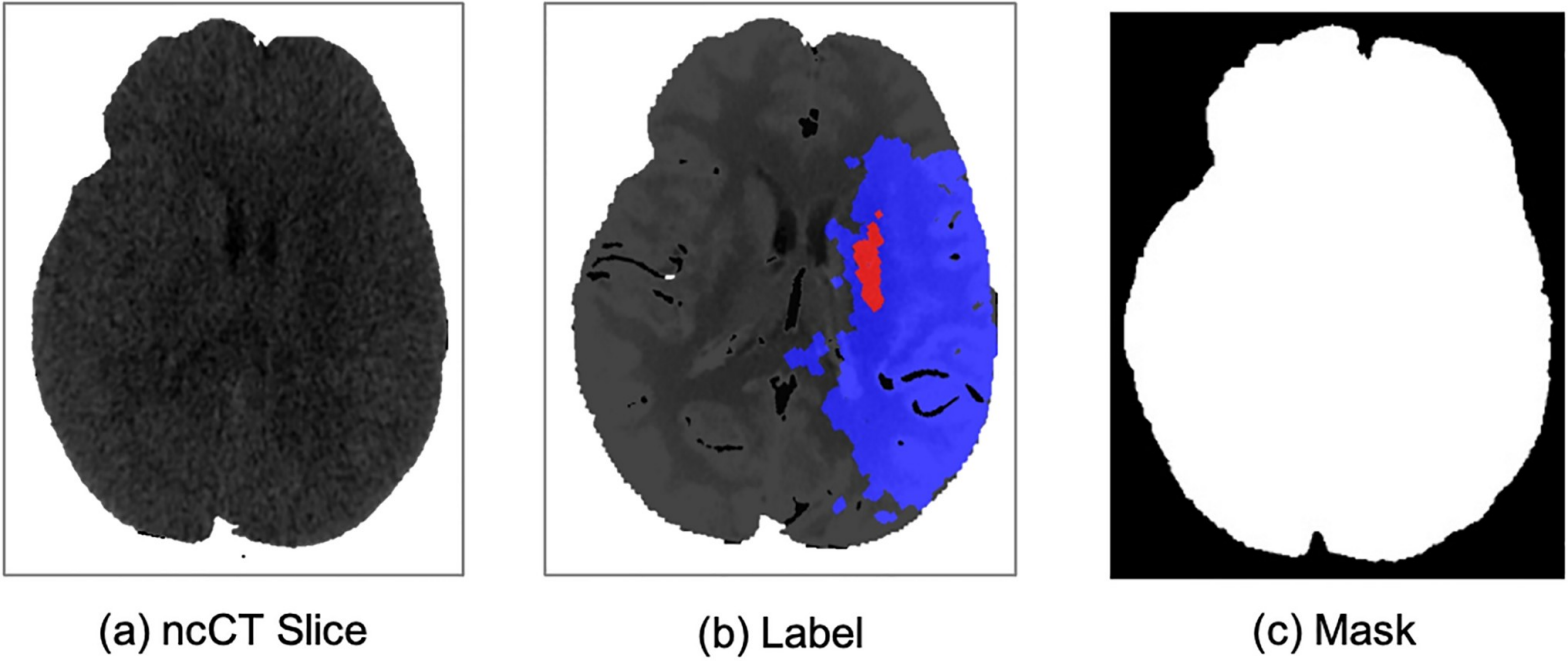
Input for feature extraction (a) brain region on an ncCT slice (b) corresponding CTp, (c) the mask of the brain region. (a) ncCT Slice. (b) Label. (c) Mask.

Fig 5 illustrates the input images for feature extraction. DeepLabv3+ is fed with *a*, *b*, and the mask *h*. The label of each input pixel is defined using *b*. The output is the feature maps and labels. The output {*F*_1_, *F*_2_, *F*_3_} is produced by CNNs MobileNet [56]v ResNet50 and ResNet101 [57] respectively, whereas *F*_4_ is generated by a special procedure designed for hand-crafted features.

#### Hand-crafted features

One of the main problems of employing ncCT images for detection of an AIS is the subtle difference between the healthy and the infarct regions. Moreover, this difference varies from patient to patient. Therefore, we propose three hand-crafted features i.e. a contralateral, local, and global features employed by a neural network. These features are combined into *F*_4_.

**Fig 6:**
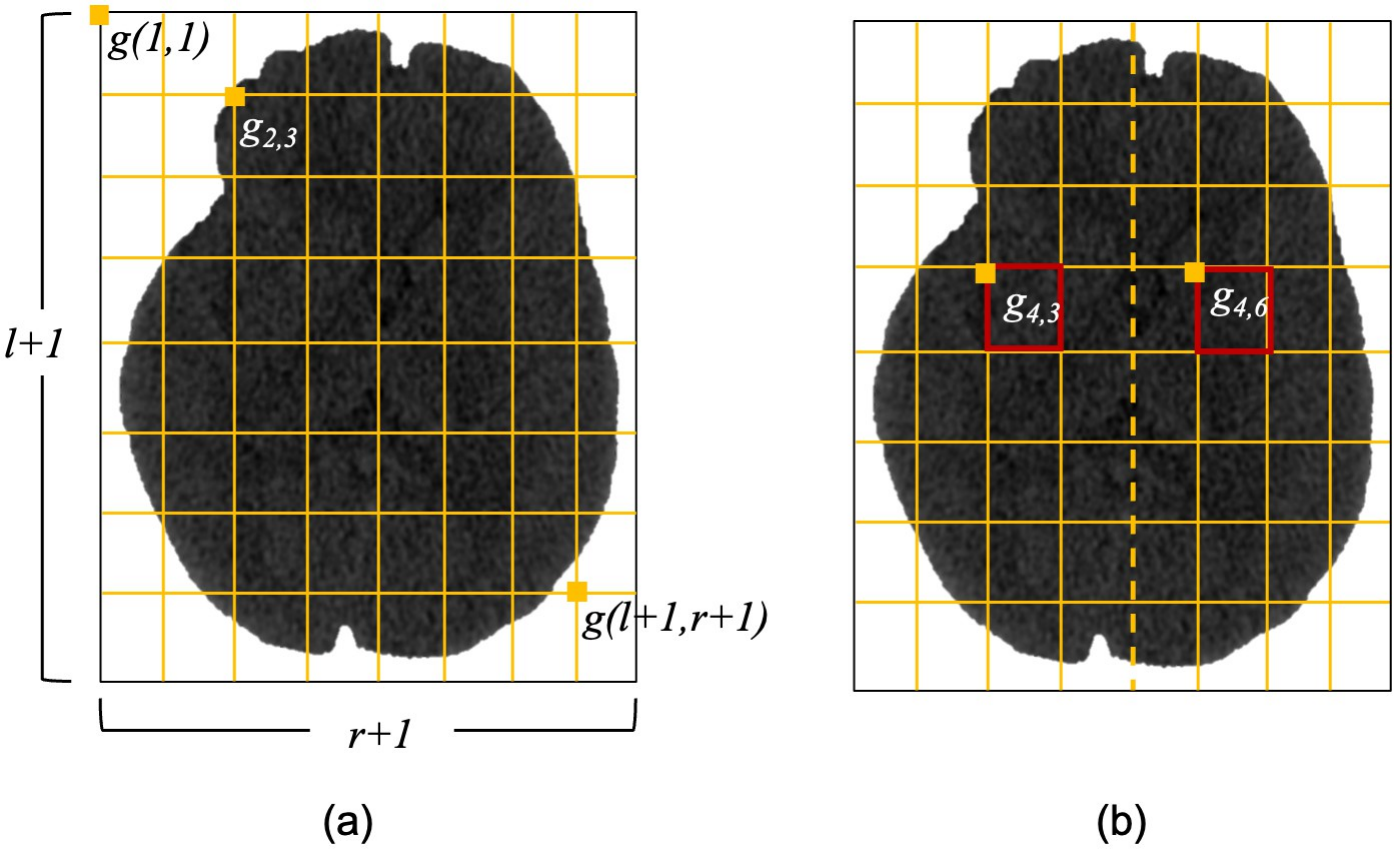
CTp and ncCT slices, and the result of aligning CTp with ncCT.

The contralateral feature, denoted by *C*, is obtained by dividing the slice into rectangular cells(Fig 6(a)). For each cell the max, mean, and median of the HU are computed. Further, for each the cell *g*(*v*, *h*). the contralateral cell *g*(*v*′, *h*′) with regard to vertical line *v* is considered. The maximum, mean, and median of pixels for each cell *g*(*v*, *h*) are stored as *C*_max_(*v*, *h*, *i*, *j*), *C*_*μ*_(*v*, *h*, *i*, *j*), $C_{\tilde{x}}\left(v,h,i,j\right)$ and ones from its contralateral cell *g*(*v*′, *h*′) are stored as *C*_max_(*v*′, *h*′, *i*, *j*), *C*_*μ*_(*v*′, *h*′, *i*, *j*), $C_{\tilde{x}}\left(v^{\prime },h^{\prime },i,j\right)$, respectively. The local feature map, *K* is generated by a sliding window. It includes the maximum, mean, median, and the is HU at the center of the window after applying the standard bilateral filter [58] i.e. *K*_max_(*i*, *j*), *K*_*μ*_(*i*, *j*), $K_{\tilde{x}}\left(i,j\right)$, *K*_*bilat*_(*i*, *j*)s. The model applies windows {8 × 8, 16 × 16, 32 × 32}. The global feature, *G*, represents the entire image using the maximum, the mean, and the median of the HU values *G*_*max*_(*a*), *G*_*μ*_(*a*), and $G_{\tilde{x}}\left(a\right)$, respectively. The pixel-wise feature *P*(*a*, *i*, *j*) (*i*, *j*) is *C*(*a*, *v*, *h*, *i*, *j*), *K*(*a*, *i*, *j*), and *G*(*a*, *i*, *j*), are concatenated. The entire feature map is then *P*(*a*, *i*, *j*) is denoted as

$$\begin{array}{cccc} P(a,i,j)= & \{ & C_{\text{max}}\left(v,h,i,j\right),C_{\mu }\left(v,h,i,j\right),C_{\tilde{x}}\left(v,h,i,j\right),C_{\text{max}}\left(v^{\prime },h^{\prime },i,j\right), & \\ & & C_{\mu }\left(v^{\prime },h^{\prime },i,j\right),C_{\tilde{x}}\left(v^{\prime },h^{\prime },i,j\right),K_{\text{max}}\left(i,j\right),K_{\mu }\left(i,j\right),K_{\tilde{x}}\left(i,j\right), & \\ & & K_{bilat}\left(i,j\right),G_{max}\left(a\right),G_{\mu }\left(a\right),G_{\tilde{x}}\left(a\right) & \} \end{array}$$

Finally, *P*(*a*, *i*, *j*) is processed by the DNN to compute the probability of a pixel to belong to the infarct area.

#### Comparison of the feature maps

**Fig 7:**
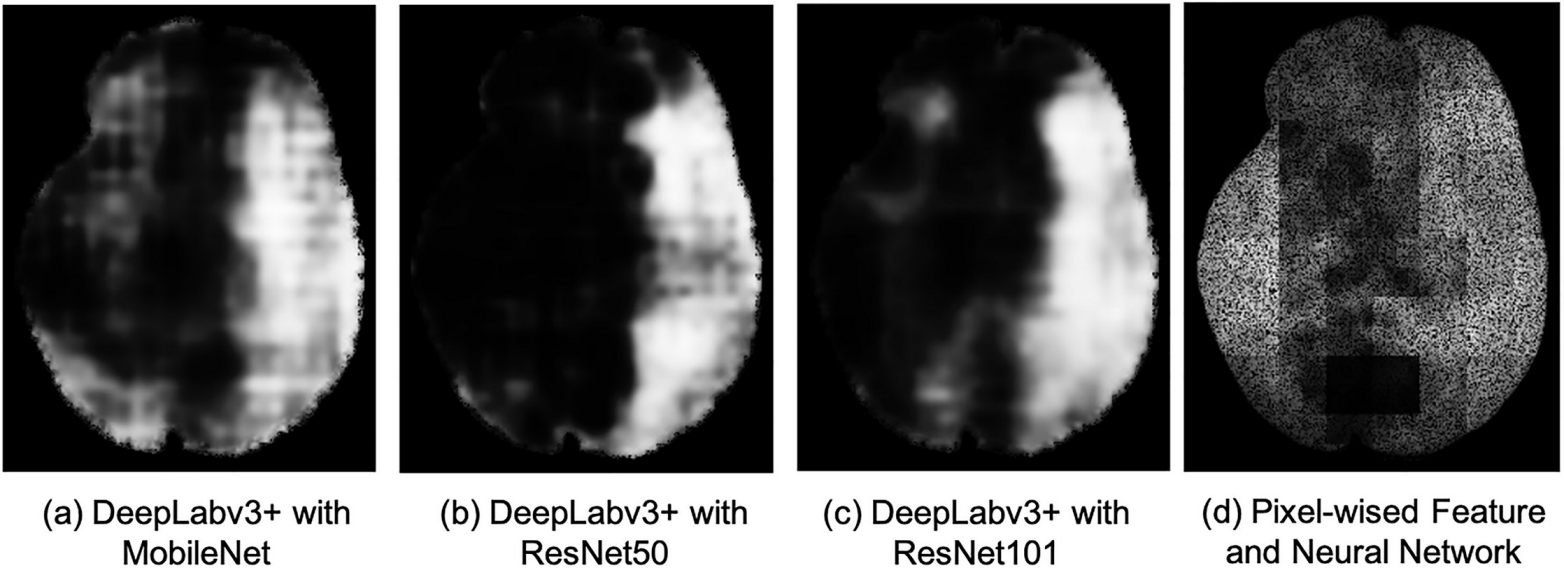
The feature maps. (a) DeepLabv3+ with. (b) DeepLabv3+ with MobileNet ResNet50. (c) DeepLabv3+ with. (d) Pixel-wised Feature ResNet101 and Neural Network.

Fig 7 illustrates the proposed feature maps. The color in the Fig represents the probability of the “infarct pixel”, where the pure white corresponds to pixels having the infarct probability 1 and black shows probability 0. The feature map extracted by ResNet50 and ResNet101 shows a clear separation of the normal and the infarct areas. However, feature map by MobileNet shows less number of disconnected regions. The output is improved by post processing.

### Infarct localization

**Fig 8:**
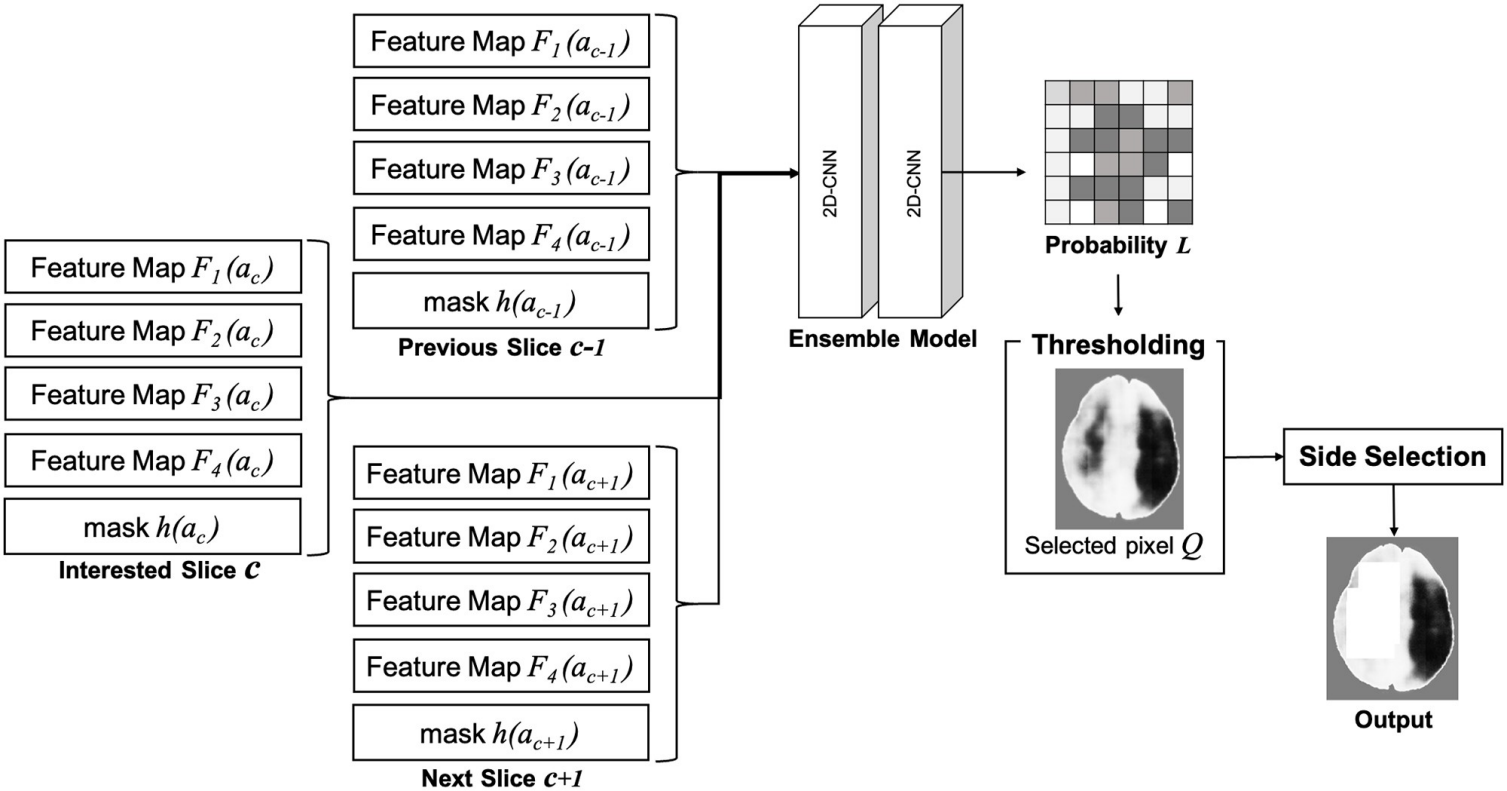
Localizing the infarct region using the extracted feature maps.

Classifying each pixel using the four feature maps is performed by an ensemble model. The probability map is post processed (see Fig 8 for illustration).

#### Ensemble model

The proposed ensemble model is designed to process multiple feature maps and utilized spatial information of the neighboring slices. It includes two CNN layers. The first layer uses 64 kernels of size 3 × 3 with 1 × 1 stride and the ReLU activation function. The input block corresponds to 1 slice and includes *F*_1_(*a*), *F*_2_(*a*), *F*_3_(*a*), and *F*_4_(*a*) of the slice *a*_*a*_. The fifth channel is the mask *h*(*a*) of the brain region. This mask is used by the first layer to manipulate the values in the region of interest. The second layer has 3 kernels 1 × 1 and with the ReLU activation function followed by a Softmax layer [59]. The output is the pixel-wise probability of the infarct. Since ensemble model is able to accept multiple input blocks we include the feature maps of the neighboring slices. Hence, the feature maps of the slice *c*, the previous slice *c* − 1 and the following slice *c* + 1 are the extended blocks of the input as depicted in Fig 8.

#### Postproceessing

The brain infarct damages often appears on one side brain. However, another side may still comprise artefacts appearing due to the noise and the subtle difference between the infarct and non infarct pixels. Therefore, the postproceessing procedure selects the left or the right side of the brain which has the largest connected region of infarct pixels.

## Experiments

The numerical experiments include 18 retrospectively analyzed fast track patients presented at Siriraj Hospital from 1 January 2017 to 31 December 2021. The age of patients is between 18 to 80. The inclusion criteria is the AIS and the availability of the sequential brain CTps. The patients with an ncCT suggestive of the acute hemorrhagic stroke were excluded. The number of ncCT-CTp pairs for each patient is 804.

### Ethical approval

Ethical approval of this study was obtained from the Ethics Committee of Siriraj Hospital, Mahidol University (416/2565(IRB3)). The need for consent has been waived due to the retrospective nature of the project and the anonymization of the metadata.

### Image acquisition

Each pair of ncCT and CTp scans was taken within 2-3 hours after the stroke onset. For each patient the ncCT and CTp scans were performed in a sequential fashion with a less than 1 hour interval between them. All patients underwent ncCT and CTp on the 256-slice Multi-Detector CT (MDCT) scanners (Revolution CT, GE Healthcare or Revolution Apex, and GE Healthcare). The procedures follow the institutional stoke-fast-track CT brain protocol. The slices are ranging from 1.25 to 5 mm.

The CTp images were obtained after the injection of 50 mL of nonionic iodinated contrast media with 350 or 370 mgI/mL (Omnipaque 350, Ultravist 370, Iopamiro 370) at 5 mL/s via an antecubital vein. Infarct regions were processed by RAPID software with a relative cerebral blood flow (rCVF) less than 30% of that in normal tissue and hypo-perfused tissue with Tmax greater than 6 seconds. The ncCT scans were acquired in axial slices continuously, with 5 mm thickness and the setting parameters were 80, 120, 140 kV, and 110-640 mA. The variation of kV and mA is due to the changing CT protocols. They establish a different number of the CT slices and a different noise index during 2017-2021. This may have an impact on the classification results. On the other hand, it is generally good for the deep learning model to be trained on a data obtained with different protocols on different machines. Finally, a minimal dataset underlying the results can be found in [60].

### Data set and performance evaluation

As previously mentioned the CTp slices are used to outline the ground truth. The pair CTp-ncCT was selected by an experiences radiologist at Siriraj Hospital of Thailand. The proposed method was tested using the leave-one-out approach. Given 18 sets of ncCT, one set of ncCT was selected for testing the rest are used for training. The procedure were repeated 18 times with 18 different testing sets. The method was evaluated quantitatively and qualitatively. The output image was sub sampled using the cells 32 × 32. The label of each cell was calculated using the majority rule. The classification performance was measured using the precision, the recall, and the F1 score defined as follows

$$\begin{array}{c} \text{precision}=\frac{\text{TP}}{\text{TP}+\text{FP}}, \end{array}$$ (1)

$$\begin{array}{c} \text{recall}=\frac{\text{TP}}{\text{TP}+\text{FN}}, \end{array}$$ (2)

$$\begin{array}{c} \text{F1}=2\times \frac{\text{precision}\times \text{recall}}{\text{precision}+\text{recall}}, \end{array}$$ (3)

where TP, FP and FN are true positive, false positive and false negative respectively. We also evaluate the results by the IoU defined as follows. Let *A* be the infarct area on the CTp slice and *B* the detected infarct area obtained by the model. The IoU is then defined by

$$\begin{array}{c} \text{IoU}=\frac{\left|A\cap B\right|}{\left|A\cup B\right|}. \end{array}$$ (4)

The higher is the value of IoU the better is tbe performance of the method.

### Quantitative evaluation

#### Feature map evaluation

The experiments were conducted using a varying size of the extended blocks of the neighboring slices i.e. 0, 3, and 5 slices. The results are shown in Table 1. The 3 slice blocks combined with postproceessing achieves the best accuracy of about 91.16%, F1 score 69.97%, precision 65.15% and recall 77.44%. Without the post the accuracy is 88.56% with 60.87% precision, 71.47% recall, and F1 score of about 65.03%. The 5 slice blocks produce are slightly lower results i.e. 90.55%, 64.66%, 76.63%, and 69.67%. The 1 slice blocks show the recall is 71.46%, and F1 score is 64.21%. The results shows that the 3 slice blocks with postproceessing provide the best combination of the accuracy, precision, recall, and F1 score.

**Table 1:** Performance of the model vs. the size of the block and post processing.

Neighbor Slice	Post Processing	Accuracy	Precision	Recall	F1 Score
0	Yes	87.47%	59.88%	71.46%	64.21%
	No	87.86%	59.04%	69.28%	62.97%
3	Yes	91.16%	65.15%	77.44%	69.97%
	No	88.56%	60.87%	71.47%	65.03%
5	Yes	90.55%	64.66%	76.63%	69.67%
	No	88.89%	62.10%	71.31%	65.61%

**Fig 9:**
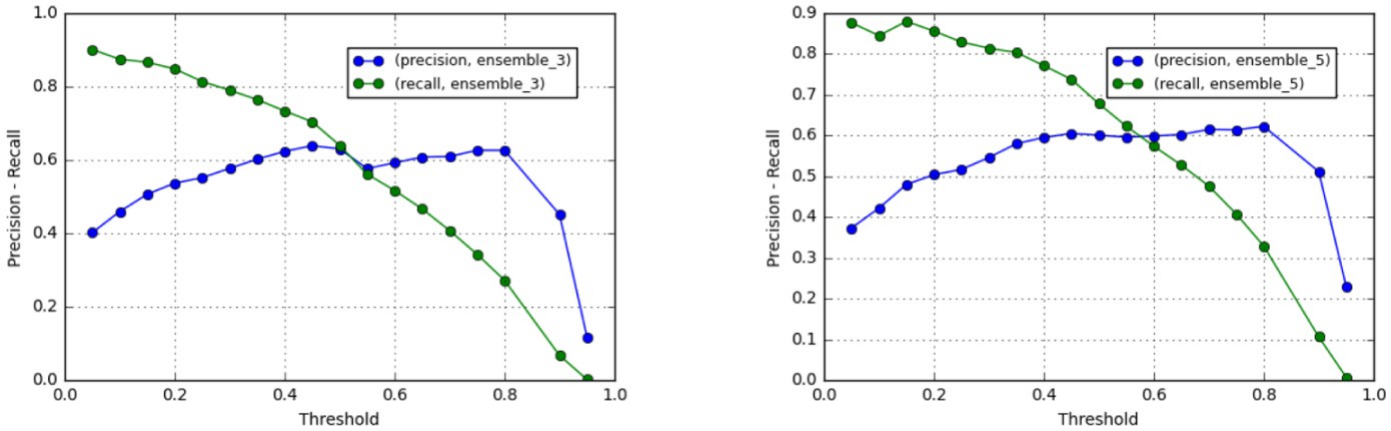
Precision and recall values of Ensemble3 (left) and Ensemble5 (right) with different classification threshold.

Comparison of precision and recall using different threshold for classifying each pixel from the probability *L* obtained from the ensemble model is shown in Fig 9. Using a precision, the threshold value of 0.5 achieved the highest precision for Ensemble3; while Ensemble5 was slightly lower.

Let us compare the performance of the DNNs applied separately. Recall that the model applies MobileNet, ResNet50 and ResNet100. The results are shown in Table 2. ResNet50 achieves the best accuracy of 86.94% along with 53.87% precision, 63.09% recall, and 56.98% F1 score. The accuracy of ResNet100 is lower but the precision, the recall, and the F1 score are higher that is 54.22%, 65.85%, and 58.69%, respectively. However, the model applying all feature maps (Ensemble0) achieves the best result. Therefore, the combination of the DNNS models improves the performance.

**Table 2:** Testing the feature maps and input blocks.

Feature maps	Post-processing	Accuracy	Precision	Recall	F1 Score
Ensemble3	Yes	91.16%	65.15%	77.44%	69.97%
	No	88.56%	60.87%	71.47%	65.03%
Ensemble5	Yes	90.55%	64.66%	76.63%	69.67%
	No	88.89%	62.10%	71.31%	65.61%
Ensemble0	Yes	87.47%	59.88%	71.46%	64.21%
	No	87.86%	59.04%	69.28%	62.97%
MobileNet	Yes	85.82%	47.94%	60.98%	53.20%
	No	84.41%	49.15%	64.13%	54.58%
Pixel-wise Feature	Yes	83.99%	48.07%	56.56%	50.38%
	No	82.04%	43.14%	55.59%	48.07%
ResNet100	Yes	85.74%	54.22%	65.85%	58.69%
	No	85.71%	52.85%	65.70%	57.80%
ResNet50	Yes	86.94%	53.87%	63.09%	56.98%
	No	84.58%	50.01%	65.38%	55.83%

**Fig 10:**
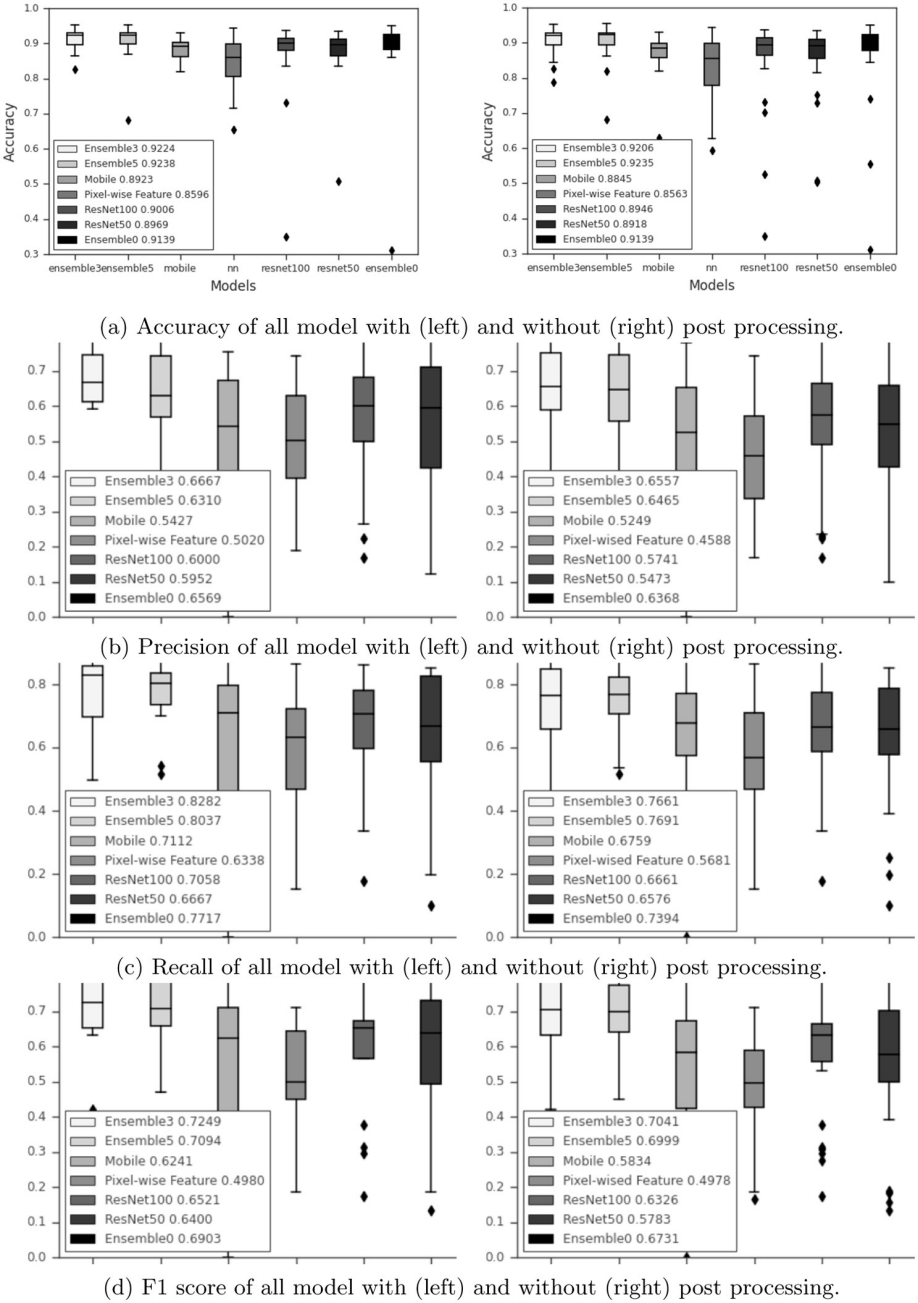
Accuracy (a), precision (b), recall (c), and F1 score (d) of Ensemble model with 3- and 5- blocks by MobileNet, ResNet101, and Resnet50. (a) Accuracy of all model with (left) and without (right) post processing. (b) Precision of all model with (left) and without (right) post processing. (c) Recall of all model with (left) and without (right) post processing. (d) F1 score of all model with (left) and without (right) post processing.

Box plots in Fig 10 show the accuracy, precision, recall, and F1 score with and without preprocessing with varying number of slices in the block. They also compare the models when only 1 DNN is applied. The number of slices in the block varies as follows. Ensemble0 uses 1 slice, Ensemble3—3 slices and Ensemble5—5 slices. The plots show the median value over the 18 runs mentioned above. The preprocessing always produces better results irrespective of the type of the back-bone DNN and the number of slices. Ensemble5 shows slightly higher accuracy, however the precision, recall and F1 are better for Ensemble3. We conjecture that 5 slices lead to the overfitted model however in general this parameter is an open problem and must be evaluated by experiments.

#### Evaluation by IoU

**Fig 11:**
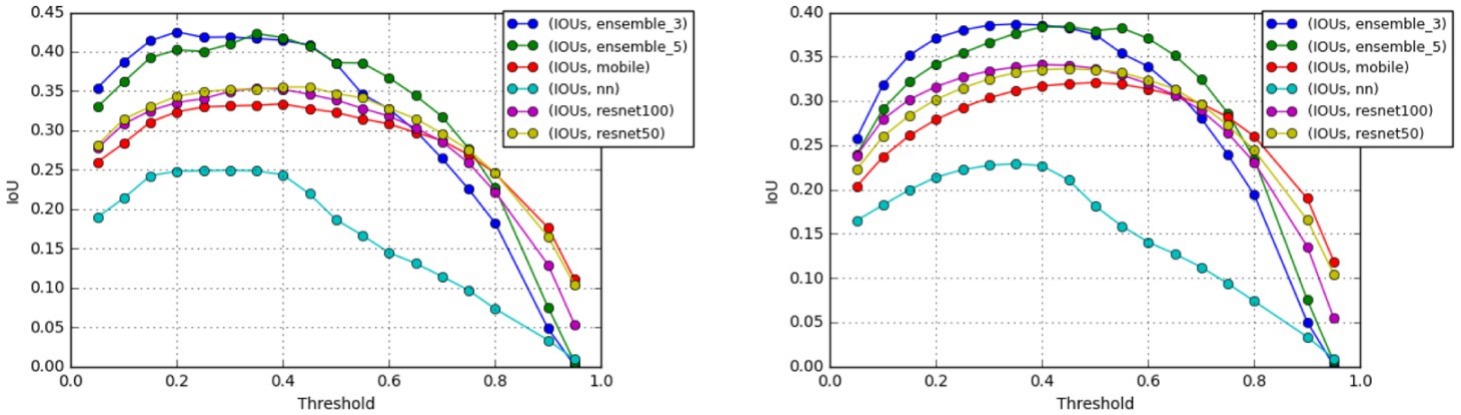
Average IoU of regions obtained from each model using different classification threshold values with postproceessing (left) and without it (right).

**Fig 12:**
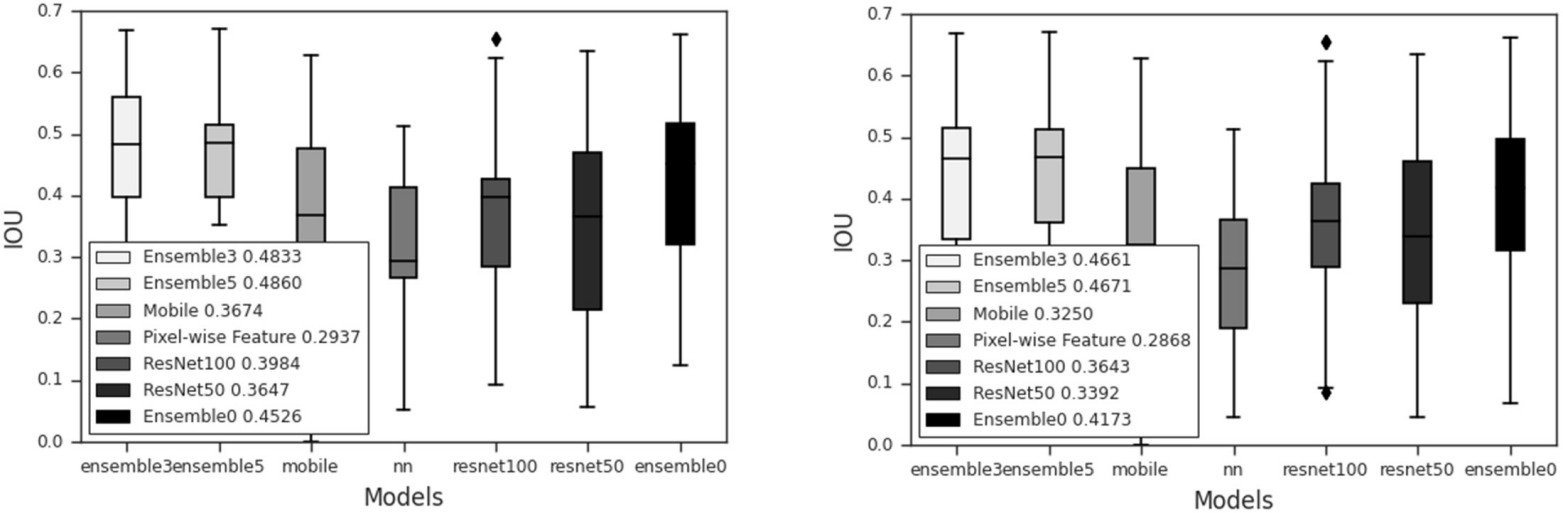
Box plots of IoU for MobileNet, hand-crafted feature map, ResNet101 and Resnet50. Ensemble model with 1, 3 and 5 extended output blocks. Each plot shows the median values. IoU of all model with (left) and without (right) post processing.

The average IoU for each of the proposed feature maps with and without post-process is shown in Table 3. postproceessing improves the IoU for every feature map. Ensemble3 and Ensemble5 achieve comparable IoUs of 0.4536 and 0.4556 respectively. ResNet100 has the highest IoU of 0.3621 while a hand-crafted map has the IoU 0.3. Ensemble0 (all features) has the IoU of about 0.413. The IoU obtained from each model with different thresholds is shown in Fig 11. The highest IoU is achieved with a threshold of about 0.5. Although employing a lower threshold shows a better IoU, many false positive lead to a low precision. A box plot of each model can be found in Fig 12.

**Table 3:** The average IoU of feature maps and input blocks.

Feature maps	Post Processing	IoU
Ensemble3	Yes	0.4536
	No	0.4002
Ensemble5	Yes	0.4556
	No	0.4082
Ensemble0	Yes	0.4131
	No	0.3820
MobileNet	Yes	0.3377
	No	0.3204
Pixel-wise feature	Yes	0.3000
	No	0.2638
Resnet100	Yes	0.3621
	No	0.3400
Resnet50	Yes	0.3548
	No	0.3385

### Qualitative evaluation

**Fig 13:**
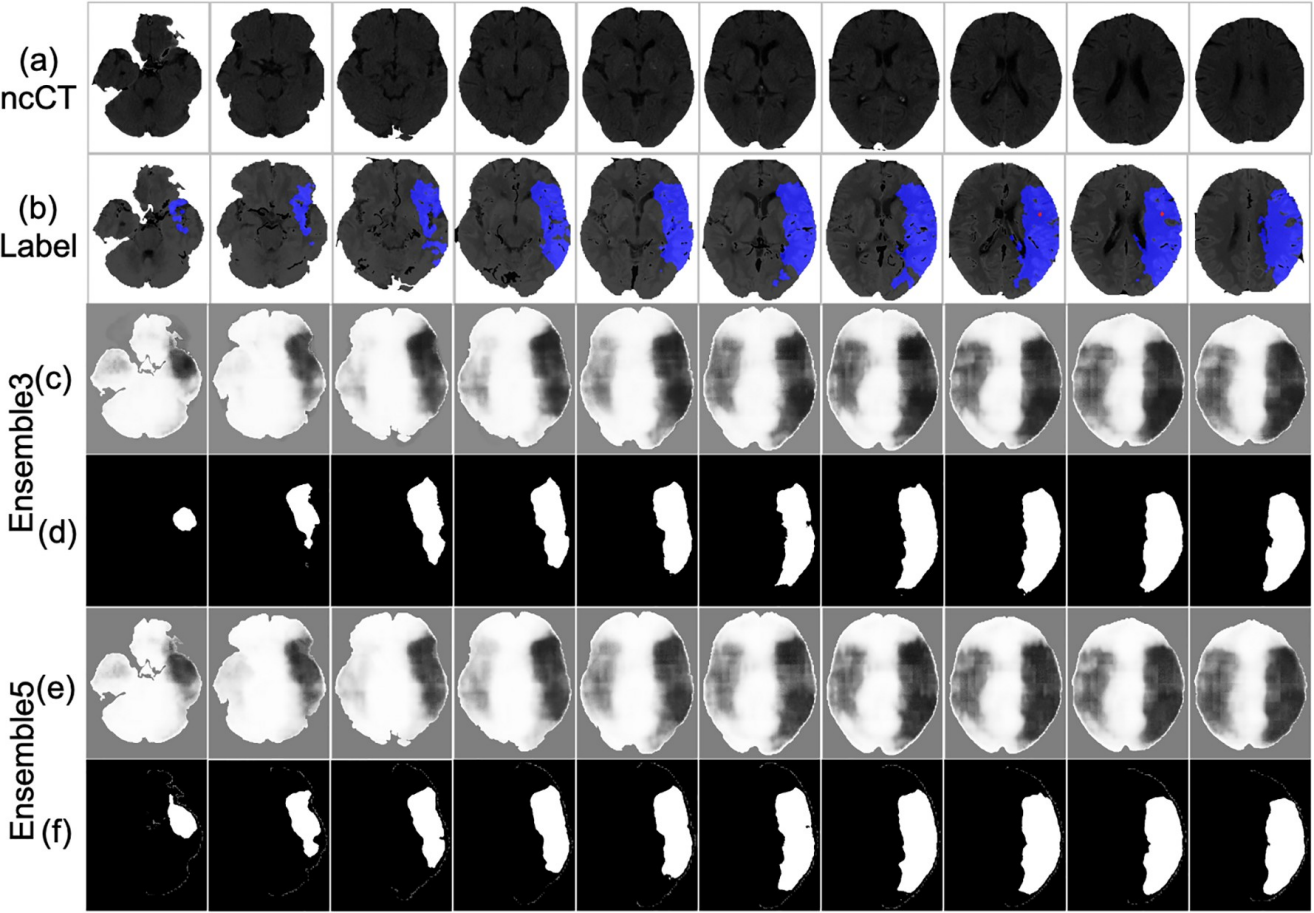
Lesion detection of the original ncCT (a) label on CTp (b) of the case that achieved the best segmentation result. (c) Ensemble3 (e) Ensemble5 without postproceessing (d) Ensemble3 (f) Ensemble5 with postproceessing.

**Fig 14:**
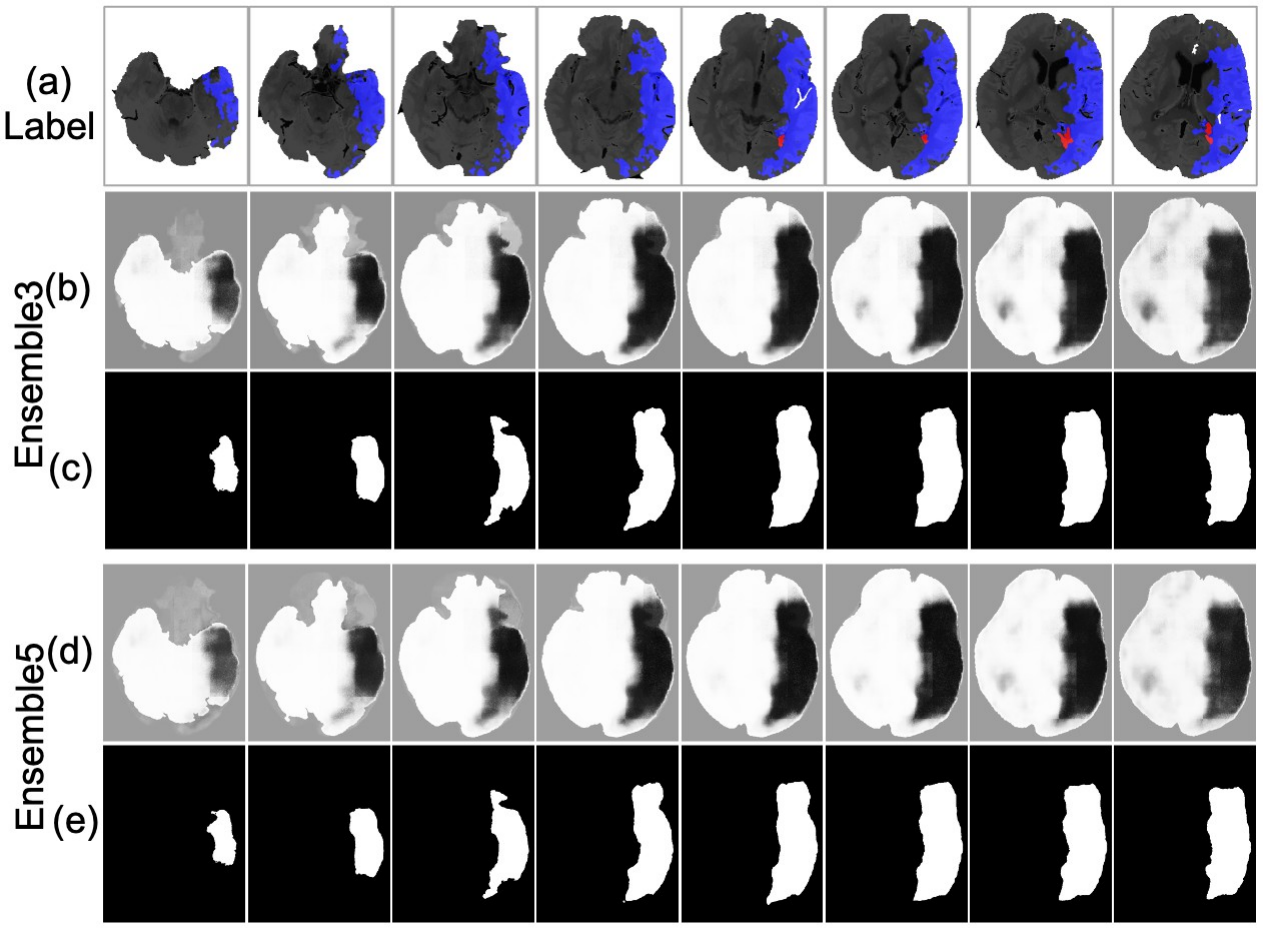
Lesion detection (a) CTp, (b), (d) Ensemble3 and Ensemble5 without postproceessing, (c), (e) Ensemble3 and Ensemble5 with postprocessing.

**Fig 15:**
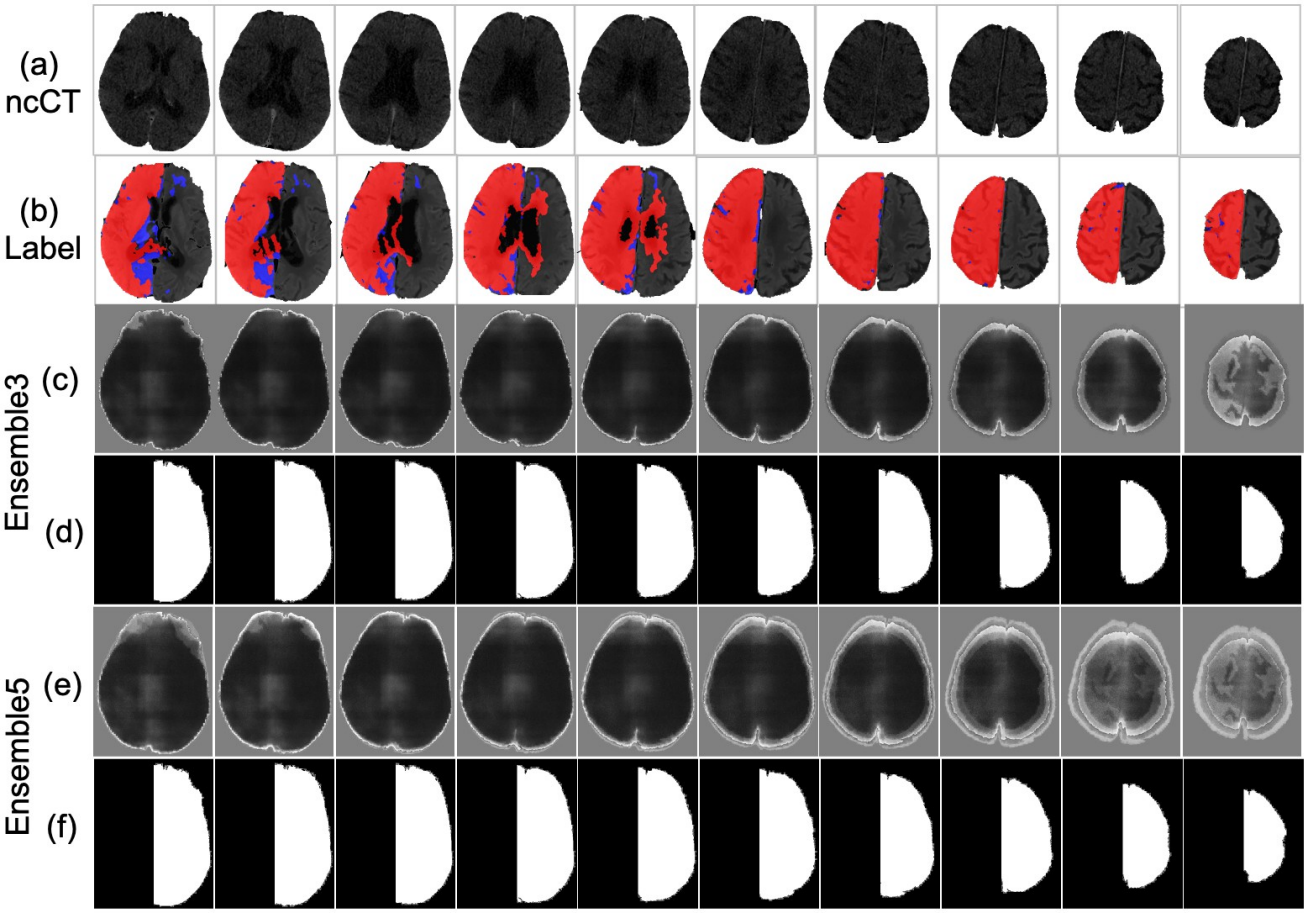
Lesion detection (a) ncCT and its corresponding CTp (b) of the case that achieved the lowest result due to the failed postprocessing, (b), (d) Ensemble3 and Ensemble5 without postprocessing (c), (e)Ensemble3 and Ensemble5 with postproceessing.

**Fig 16:**
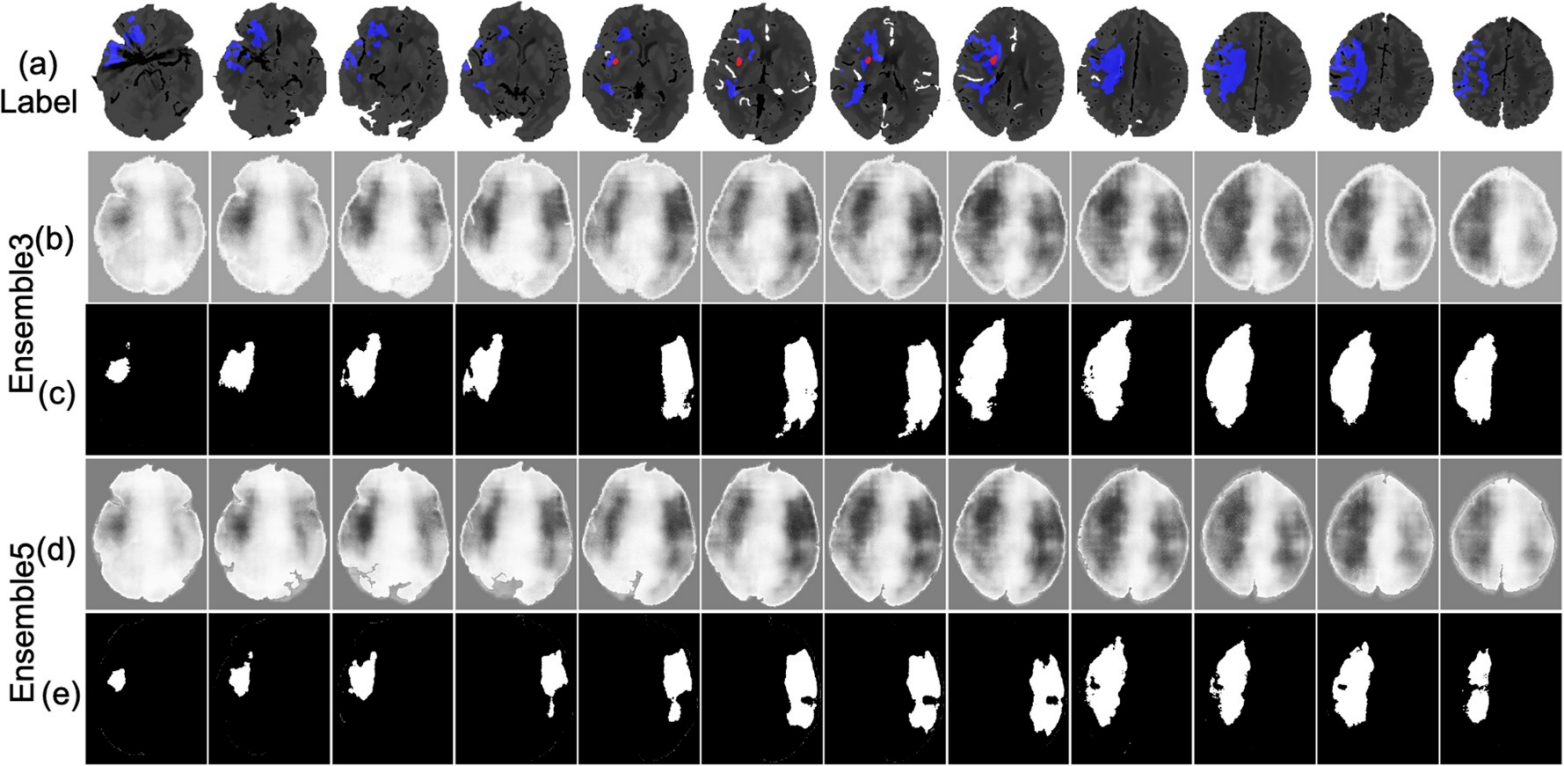
Lesion detection of the label on CTp (a) that achieve the low result. The result by Ensemble3 and 5 without postproceessing is depicted in (b) and (d), respectively. The result with postproceessing is in (c) and (e) respectively.

An example of the infarct detection characterized the highest classification measures is shown in Fig 13. The lesion obtained from the Ensemble3 and Ensemble5 is shown in Fig 13(c) and 13(d). Visually the models correctly locate the infarct area. The accuracy of 94.14% and 92.96% is achieved by Ensemble3 and Ensemble5 respectively. The postprocessing also improves the IoU of Ensemble3 from 0.4645 to 0.5153 and Ensemble5 has been improved from 0.4719 to 0.5117. The result is the average over the 804 slices. Fig 14 shows the segmentation by Ensemble3 that achieves the highest IoU of 0.6401 and 92.90% accuracy. Whether the postproceessing was applied or not, the Ensemble5 has the same accuracy of 92.88% and IoU of 0.6427 The case with the lowest accuracy is depicted in Fig 15. The model can not detect the correct side of brain. This is a single case in the dataset. Fig 16 shows the case when results for the Ensemble3 and 5 are different i.e. Ensemble3 selects 3 slices on the left whereas Ensemble5 has them on the right. The accuracy of Ensemble3 and Ensemble5 without postproceessing is 91.01% and 91.18%. However with postproceessing Ensemble3 and Ensemble5 produce 87.50% and 91.72%. Therefore, for some cases when the postproceessing fails on some slices the increase of the size of the training block may improve the accuracy.

## Discussion

Computerized image analysis developed using machine learning and artificial intelligence is applicable to interpret and assess the ncCT images of the AIS. Preprocessing for aligning CTp obtained from an automated imaging software RAPID with the ncCT is useful for validation. The proposed ensemble model is able to detect the brain infarct area on the ncCT. The method has been tested on early stroke patients using the leave-one-out cross validation. The model achieves the average accuracy of 91.16% and 77.44% recall (sensitivity). The proposed postproceessing improves the average IoU measure from 0.4 to 0.45. Though the automated CTp software RAPID has been validated and used in many hospitals the cost of software is high. Therefore, it may not be affordable in many countries. Therefore the proposed application could have a critical impact on the accuracy of detection and localization of the infarct in clinical conditions. There are limitations of this study. It has been performed at a single clinical center and is retrospective. The selection of the samples is biased. The number of cases is relatively small and the parameters of the CT scanners are not standardized. This includes the number of the ncCT slices and the noise. Note that the data variation is not necessarily bad for AI learning. However, the effects of these variations are not clearly understood. Therefore, the paper reports preliminary results. Further studies which include the data from different clinical facilities are to be performed. In particular further studies to calculate the ASPECTS score could improve applicability of the method.

## Conclusions

The proposed ensemble model employing end-to-end feature maps is able to detect the brain infarct area on the ncCT of the stroke patients. The tests against the automated CTp software show a favorable outcome. Further prospective studies which include the data from different clinical facilities could confirm these preliminary results.
